# Multiscale Comparative Analyses of OVB-Organoids and Cerebral Organoids

**DOI:** 10.3390/cells15080703

**Published:** 2026-04-16

**Authors:** Jingyi Yang, Xue Zhang, Shuang Li, Zhixian Gao

**Affiliations:** Tianjin Key Laboratory of Risk Assessment and Control Technology for Environment and Food Safety, Military Medical Sciences Academy, Academy of Military Sciences, Tianjin 300050, China; jingyiyang_959@163.com (J.Y.); zhang_jn0307@163.com (X.Z.)

**Keywords:** OVB-organoids, cerebral organoids, organoid culture method, in vitro models

## Abstract

**Highlights:**

**What are the main findings?**

- Standardized protocols for cerebral organoids and OVB-organoids were established; multiscale analyses identified core differences in their cellular composition, structure, function and developmental trajectories.
- OVB-organoids outperform cerebral organoids in simulating early retinal development and visual-related functions, with specific VSX2 expression and transcriptomically consistent gene profiles.

**What are the implications of the main findings?**

- OVB-organoids, as an improved in vitro model, better simulate early neurodevelopment and provide a reliable basis for clarifying early eye–brain coordinated development mechanisms.
- The standardized OVB-organoid model has broad application prospects and optimizes the neurodevelopment organoid system, offering a new platform for eye–brain development research.

**Abstract:**

Organoid technology is critical for studying human brain development, but existing single-organoid culture systems fail to simulate inter-organ interactions (e.g., optic vesicle–brain) in embryogenesis. This study aimed to establish a more comprehensive in vitro model for early neurodevelopment. We established standardized protocols for cerebral organoids and optic vesicle-containing brain organoids (OVB-organoids), and performed multiscale analyses using immunofluorescence and transcriptomics to compare the two models. Key differences in cellular composition, structure, function and development were found; OVB-organoids better simulated early retinal development, visual-related structures/functions, with specific VSX2 expression and consistent transcriptomic profiles. OVB-organoids are an improved model for early neurodevelopment, providing a reliable basis for exploring eye-brain coordinated development mechanisms and having broad applications in developmental biology, disease research and personalized healthcare.

## 1. Introduction

Organoids have garnered significant attention in recent years as in vitro models constructed from stem-cell-derived three-dimensional (3D) cell aggregates using in vitro 3D culture techniques [1]. The genetic inheritance, cell proliferation and differentiation of organoids are closely related to stem cell populations and exhibit characteristics, such as cell self-renewal, cell self-assembly, organ physiological activity, long-term culture and genetic stability. Organoids resemble the physiological state of in vivo organs more closely than traditional in vitro models (cell and animal models) [2]. Therefore, organoids have vast potential for applications in disease modeling, drug development, and precision medicine at the organ level, making them ideal carriers for future research in life sciences [3].

In 2009, Clevers et al. [4] successfully initiated an era of organoids by cultivating small intestinal crypt and villus structures from adult stem cells in vitro. Various organoids have been developed to simulate or replicate the main features of organ development in the brain [5,6,7], intestine [8,9], liver [10,11], heart [12,13], breast [14,15], lung [16,17], retina [18,19] and skin [20,21]. Organoid models are widely applied in disease modeling [5,20], drug screening [11,12], precision medicine [8,9], gene therapy [22,23], regenerative medicine [19] and exposure assessment [24] (Table 1). Zhu et al. [25] established a hypoxic stroke model using human-induced pluripotent stem cell (hiPSC)-derived brain organoids to simulate hypoxic stroke caused by ischemia. The mechanism of injury was studied using proteomics, single-cell transcriptome and histopathological analyses. They identified a possible role of a traditional Chinese medicine formula called the ‘DengZhanShengMai’ capsule in ischemic and hypoxic stroke treatment through the regulation of lipid metabolism-related biological functions. Kwak et al. [26] introduced a novel epidermal construct, epidermoid organoids, derived from induced pluripotent stem cells (iPSCs), which can produce effective extracellular vesicles (EVs) for skin regeneration. These EVs contribute to target cell proliferation, migration, and angiogenesis, providing a promising therapeutic technique for wound healing and an efficient model for skin research and future therapeutic applications.

In complex physiological environments, most physiological functions and diseases are interconnected and are mutually influenced by multiple organs. Research has shown that single independent organoids cannot adequately reflect this synergistic interaction. Therefore, with the continuous development of organoid technology, the reproduction of connections between multiple organs has gradually become a new research focus. Multi-organoid fusion culture systems have been preliminarily investigated and established (Table 2). Martins et al. [27] induced neuromesodermal progenitors from pluripotent stem cells (PSCs) to simultaneously generate spinal motor neurons and skeletal muscle cells, which spontaneously assemble into neuromuscular organoids (NMOs) in a 3D culture. NMOs contain functional neuromuscular junctions, making this model capable of simulating myasthenia gravis and demonstrating significant potential for modeling neuromuscular diseases. Yin et al. [28] generated the first self-organizing neuromuscular skeletal organoids from PSCs using a co-developmental strategy. This approach achieved the co-differentiation and spatial self-organization of three types of tissues, resulting in a composite organoid containing three interconnected regions, thereby providing a human-derived in vitro model for studying related diseases.

Human brain organoid models have attracted significant attention because of their specific advantages in neurodevelopmental research owing to the differences in the formation of cortical loops between humans and rodents [29]. Research on brain organoids can be traced back to 2013, when Lancaster et al. [30] successfully derived 3D cultures with brain-like structural characteristics from PSCs under suspension conditions and named them ‘cerebral organoids.’ Although whole-brain organoids contain various brain-region cells, the application of non-directed induction limits the formation of complex brain regions [31]. Therefore, region-specific brain organoids, such as cortical organoids [32], thalamic organoids [33], ventral forebrain organoids [34], and hippocampal organoids [35], have been successfully cultured. The co-culture of brain organoids with organoids from other tissues, as well as organoids with distinct regional characteristics [36], is also a new research direction to enhance the simulations of the physiological features of the brain, interactions between different brain regions, and regulatory relationships between the brain and other organ systems [37]. The optic vesicle-containing brain organoid (OVB-organoid) is a unique brain organoid that integrates the structure of the ‘optic vesicle’ (which can form the retina) into the brain organoid, facilitating the examination of the close physiological correlation between visual function and the brain. During embryonic development, the optic vesicle evolves from the diencephalon and is the eye primordium attached to the forebrain. The OVB-organoids are derived from iPSCs and are characterized by their ability to spontaneously develop bilaterally symmetric optic vesicles from the anterior region of the brain-like area, demonstrating the inherent self-patterning capability of iPSCs during highly complex biological processes. OVB-organoids can simulate eye–brain interactions, model congenital retinal diseases, and generate specific retinal cells for personalized drug testing and transplantation therapies more comprehensively than ordinary brain organoids.

The aim of this study was to conduct multiscale comparative analyses of OVB-organoids and cerebral organoids. First, we cultured cerebral organoids and OVB-organoids derived from iPSCs. Using a series of comprehensive characterization methods, we then conducted a detailed analysis of the two organoid models in multiple dimensions to reveal their differences in terms of morphology, histology, protein expression, and gene expression. Using transcriptomics as a foundation, we systematically analyzed the gene expression profiles of the two organoids. To clarify the biological functions of these differentially expressed genes (DEGs), we performed Kyoto Encyclopedia of Genes and Genomes (KEGG) and Gene Ontology (GO) enrichment analyses to identify the regulatory networks of the signaling pathways and cellular functional modules involved. These findings reveal the developmental mechanisms of the two organoids and significant features of their biological functions. Through comparative analysis, we comprehensively assessed and compared the biological characteristics and potential applications between OVB-organoids and cerebral organoids. The findings of this study provide a preliminary theoretical basis for model selection in disease research, drug screening, and regenerative medicine studies.

**Table 1:** Classification of existing partial organoid models and their applications in biomedical research.

Type	Applications	Fields	Reference
brain organoid	Screening and identification of developmental defects in autism using the CRISPR–human organoid–scRNA-seq (CHOOSE) system identifies developmental defects in autism	Neurodevelopmental Disorders Research	[5]
brain organoid	Neurofunctional repair post-brain injury via innovative Organoid–Brain–Computer Interfaces (OBCIs) promoting neural regeneration through implanted brain organoids	Regenerative Medicine	[38]
liver organoid	Drug-induced liver injury (DILI) risk prediction using high-throughput human liver organoid screening	Drug Screening, Drug Development, Personalized Medicine	[10]
liver organoid	In vitro human liver fibrosis modeling	Disease Modeling	[11]
intestinal organoid	Functional small intestinalized colon (SIC) generation via ileal-derived organoid replacement for short bowel syndrome (SBS) therapy	Regenerative Medicine	[8]
cardiac organoid	Dynamic assessment of polystyrene nanoplastics (PS-NPs) cardiotoxicity via cardiac organoid-on-a-chip (COoC)	Toxicological Assessment	[39]
skin organoid	Efficient minimally invasive hair follicle organoid delivery for biomimetic hair regeneration using gelatin methacrylate cryo-microneedles (GelMA-cryoMN)	Organ Regeneration	[40]
breast organoid	Personalized therapy guidance for advanced breast cancer through patient-derived organoids	Personalized Medicine	[14]
lung organoid	Pulmonary nanomaterial-driven response prediction with lung organoids	Toxicological Assessment	[16]
retinal organoid	Retinitis pigmentosa (RP) treatment via retinal organoid transplantation	Organoid Transplantation, Disease Therapy, Regenerative Medicine	[19]

**Table 2:** Established multi-organ organoid fusion culture strategies and their applications in biomedical research.

Type	Culture Strategy	Applications	Fields	Reference
Neuromuscular organoids (NMOs)	Self-assembly fusion of iPSC-derived spinal neurons and skeletal muscle cells	Myasthenia gravis modeling	Disease modeling	[27]
Neuromusculoskeletal tri-tissue organoids	Co-development strategy enabling simultaneous differentiation and spatial self-organization of three tissue types	Modeling human neuromuscular skeletal tissue interactions, disease mechanisms, and drug discovery	Disease modeling, Drug development	[28]
Human hepato-biliary-pancreatic organoids	Continuous patterning: Differentiation of iPSCs into foregut/hindgut spheroids followed by fusion-induced boundary self-patterning	Platform for studying human development, congenital diseases, drug development, and therapeutic transplantation	Disease modeling, Drug development, Therapeutic transplantation	[41]
Multi-organoid chip (human liver-islet axis)	Co-culture of hiPSC-derived hepatic/islet organoids under circulatory perfusion	Investigating insulin/glucose regulation in physiological and diseased states	Pathogenesis research, Drug evaluation	[42]
Vascularized macrophage-islet (VMI) organoids	Co-culture of hiPSC-derived β cells, endothelial cells, and macrophages	Modeling immune-mediated pancreatic β-cell pyroptosis post-viral infection	Disease modeling, Viral injury mechanism research	[43]
Vascularized renal organoids	3D assembly of hPSC-derived endothelial-like organoids with renal organoids	Novel model for assessing renal differentiation and cellular morphogenesis	Personalized medicine	[44]
Vascularized brain organoids	Fusion culture of mesoderm/neuroectoderm-derived organoids at specific developmental stages	Elucidating intercellular interactions during brain development	Human development research	[45]
Fused forebrain dorsoventral organoids	Spontaneous fusion of iPSC-derived dorsal/ventral forebrain organoids via signaling modulation	Modeling interneuron saltatory migration and neurodevelopmental disorders	Neurodevelopment modeling, Neurodevelopmental disorder research	[46]
Tumor-brain organoids (TBO)	Incorporation of embryonal tumor cells into hiPSC-derived forebrain organoids	Investigation of tumor biology, tumor–neural microenvironment interactions, and high-throughput drug/toxicity screening in pediatric brain tumor precision oncology	Drug screening, Toxicological assessment	[36]

## 2. Materials and Methods

**Reagents and materials:** All reagents, antibodies, and materials used in this study for the maintenance, differentiation, and organoid culture of iPSCs, as well as for the characterization of cells and organoids, are detailed in Table S1 (Reagents and Materials for iPSC and Organoid Culture and Characterization) of the Supplementary Materials.

**Generation of cerebral organoids:** The overall experimental methods are adapted from the literature [47], with slight modifications.

**Feeder-dependent hiPSC lines:** HiPSCs were cultured in a standard incubator (37 °C, 5% CO_2_). Six-well plates were coated with 1% Matrigel^®^ (Corning Inc., New York, NY, USA) (1 mL/well) for at least 2 h under the same conditions. The hiPSCs were seeded onto coated plates and maintained in a mTeSR1 medium, and the medium was replaced every two days. At 70–80% confluence levels, cells were gently dissociated into small aggregates using 0.5 mM EDTA (in Ca^2+^/Mg^2+^-free DPBS) and passaged to freshly coated plates. During the initial seeding process, the cultures were supplemented with 10 µM Y27632 (ROCK inhibitor Selleck Chemicals LLC, Houston, TX, USA) to enhance cell viability, as previously reported [48,49].

**Production of embryoid bodies (EBs) and initiation of germ layer differentiation:** At 80% iPSC confluence, the cells were washed with Ca^2+^/Mg^2+^-free DPBS. The solution was discarded, and the cells were incubated with 0.5 mM EDTA for 4 min at 37 °C. After removal, the cells were treated with Accutase for another 4 min. Digestion was terminated with mTeSR1 medium, centrifugation (270 *g*, 5 min) was applied, and single cells were re-suspended in a low-bFGF medium containing 50 µM Y27632. The concentration was adjusted to 6.0 × 10^4^ cells/mL, and the cells were transferred to round-bottom low-adhesion 96-well plates (150 µL/well) and cultured to form EBs. We replaced 50% of the medium with 150 µL fresh low-bFGF medium (supplemented with 50 nM Y27632 and 4 ng/mL bFGF) every 48 h. When the diameters of the EBs reached 350–600 µM, the medium was exchanged every 48 h with a basal low-bFGF medium (without Y27632 and bFGF).

**Induction of primitive neuroepithelia:** On post-aggregation day 6 (EB diameters: 500–600 μm), the EBs were transferred to low-adhesion 24-well plates. The medium was discarded, and 500 μL neural induction medium of cerebral organoid (NIM-cerebral organoid) was added. After 48 h, an additional 500 μL of NIM-cerebral organoid was supplemented. Ectodermal differentiation was monitored via microscopic identification of peripherally translucent zones around the EBs. Neural epithelial specification was performed when these zones developed the radial architecture characteristics of a pseudostratified neuroepithelium.

**Transfer of neuroepithelial tissues to Matrigel^®^ droplets:** A microwell template was prepared by molding a sterilized sealing film in a 100 mm diameter Petri dish. The neural epithelial tissues were individually transferred into microwells using fire-polished wide-bore tips (1.5–2 mm opening). The spent medium was aspirated, and each tissue was immediately overlaid with 30 µL Matrigel^®^. The tissues were incubated at 37 °C for 30 min for polymerization. Subsequently, a 60 mm diameter Petri dish containing 5 mL cerebral organoid differentiation medium without vitamin A was prepared. The sealing film with the embedded tissue was carefully transferred into a new dish for suspension culture.

**Stationary culture of expanding neuroepithelial buds:** At 24 h post-embedding, the tissues were examined under a microscope. Neuroepithelial buds typically emerged, exhibiting expanded pseudostratified structures with ventricle-like lumens within 1–3 days. After an additional 24 h, the medium was replaced with a 5 mL fresh differentiation medium.

**Growth of cerebral tissue:** After four days of static culture, the embedded organoids were transferred to new 100 mm diameter Petri dishes for dynamic culture on an orbital shaker (57 rpm). At this stage, cultures were supplemented with 15 mL of vitamin-A-enriched differentiation medium and replaced every 3–4 days until Day 30.

**Generation of OVB-organoid:** The overall experimental methods are adapted from the literature [50], with slight modifications.

**Cultivation of hiPSCs:** The cultivation method is as described in the subsection ‘Feeder-dependent hiPSC lines’, in which the mTeSR Plus medium was used instead of the mTeSR1 medium, and ReLeSR was utilized for cell dissociation.

**Neural induction:** At 70–80% hiPSC confluence, the cells were dissociated into single cells for neural induction. The cells were washed three times with phosphate buffered saline (PBS) and incubated with the addition of the cell dissociation reagent, Accutase (37 °C, 5 min). Digestion was terminated with the neural induction medium of OVB-organoid (NIM-OVB-organoid) containing 10 μM Y27632. Centrifugation (300× *g*, 3 min) was performed, the supernatant was discarded, and the cell precipitation was resuspended in fresh NIM-OVB-organoid with 10 μM Y27632. The density was adjusted to 1.0 × 10^5^ cells/mL, 100 μL/well was aliquot into 96-well v-bottom low adherent plates. The plates (300× *g*, 3 min) were centrifuged to aggregate the cells, incubation was conducted, and 50% of the fresh NIM-OVB-organoids was replaced daily.

**Neurospheres in Petri dishes:** On Day 5 of neural induction, neurospheres were transferred from the 96-well plates to 100 mm diameter Petri dishes at a density of approximately 50 neurospheres per dish. The NIM-OVB-organoids were discarded and replaced with 10 mL of the prepared neurosphere medium (Table S2). After 72 h, 2 mL of OVB-organoid medium (Table S3) was added to each dish. After an additional 48 h, the medium was thoroughly replaced with the OVB-organoid medium (10 mL/dish). Subsequently, the Petri dishes were transferred to an orbital shaker at 65 rpm for dynamic culture to enable the neurospheres to develop into OVB-organoids.

**Cryosectioning of organoids:** Organoids were immersion-fixed in 4% paraformaldehyde (PFA) for 30 min at room temperature (RT) to preserve cellular architecture and protein integrity. Next, the organoids were washed (3 × 5 min) with PBS to eliminate residual PFA. Fixed organoids were then sequentially immersed in sucrose solutions for cryoprotection: 10% (1 h), 20% (6 h), and 30% (12–16 h/overnight) at 4 °C, until the organoids sank to the bottom of centrifuge tubes. Dehydrated organoids were embedded in an optimal cutting temperature compound (Tissue-Tek^®^ O.C.T.) and cryo-sectioned at a thickness of 10 μm with Cryostat. Frozen specimens were mounted onto adhesive slides, dried for 30 min at RT, and preserved for long-term storage at 80 °C.

**Immunofluorescence microscopy:** Frozen sections were thawed at RT and dried naturally. First, the sections were immersed in PBS for 10 min, placed in preheated sodium citrate buffer (95 °C), and heat at 95–100 °C for approximately 15 min. Subsequently, the samples were cooled to RT and washed with PBS (3 × 5 min). This procedure involved antigen retrieval from the tissue sections, which aided in exposing the fixed antigen epitopes and improving antibody-binding efficiency. Next, the sections were treated with 0.5% Triton X-100 solution for 15 min for permeabilization to increase cell membrane permeability to antibodies. The sections were then washed with PBS (3 × 5 min) and blocked with 10% goat serum for 1 h to reduce non-specific binding. After blocking, primary antibodies diluted in the blocking solution were applied to sections, and the sections were incubated overnight at 4 °C (SOX2:1/200; PAX6:1/300; Nestin: 1/50; MAP2:1/50; Ki67:1/250; TBR2:1/100). The next day, after adjusting the temperature of the sections to RT, unbound primary antibodies were washed off with PBST (3 × 5 min), and fluorescent secondary antibodies diluted in blocking solution [Goat Anti-Rabbit IgG H&L (Alexa Fluor^®^ 488): 1/800; Goat Anti-Mouse IgG H&L (Alexa Fluor^®^ 594): 1/800] were added. This process was followed by incubation in the dark at RT for 2 h. Finally, the nuclei were counter-stained with DAPI after washing with PBST (3 × 5 min), followed by washing with PBST (3 × 5 min) to remove excess DAPI. After drying the coverslips, fluorescent signals were observed and recorded using fluorescence microscopy (Ex/Em: AlexaFluor^®^ 488). Three sections were stained per group, three random fields from each section were selected for image capturing, and image analysis was performed using the ImageJ software (Version 1.54g). The obtained semi-quantitative results were statistically analyzed using GraphPad Prism 9 software. The statistical analysis was based on three independent experiments. Two-tailed independent sample *t* test was used to evaluate the differences between data groups. When *p* < 0.05, defined as statistically significant.

**RNA sequencing:** RNA sequencing and data analysis were conducted using the DNBSEQ platform from BGI (Shenzhen, China) following established protocols. Briefly, total RNA was extracted from the collected organoids, and RNA quality was validated (concentration, purity, and integrity). The RNA was enriched and fragmented, and first-strand cDNA was synthesized using random hexamer primers and reverse transcriptase. The second-strand cDNA was synthesized with dUTP labeling to construct a strand-specific library. End repair and adapter ligation were performed, followed by PCR amplification using a high-fidelity DNA polymerase. The quality of amplified DNA was assessed in terms of fragment size and concentration. Finally, high-throughput sequencing was performed on an Illumina NovaSeq 6000 platform, and the DESeq2 (v1.10.1) package was used to examine DEG expression. A gene was considered differentially expressed if statistically significant differences in gene expression (q-value < 0.05) and a fold change >2 were observed.

**Real-time quantitative polymerase chain reaction (RT-qPCR):** Total RNA was extracted from the organoids using the RNAsimple Total RNA Kit (TIANGEN Biotech (Beijing) Co., Ltd., Beijing, China). Specific procedures included cell lysis, sample pre-processing, RNA precipitation and adsorption, protein removal and washing, and RNA elution and storage. After quality assessment of extracted total RNA, reverse transcription was performed using PrimeScript™ RT Master Mix (Takara Bio Inc., Shiga, Japan) to generate cDNA. The 20 μL reaction system comprised 10 μL 2× PrimeScript™ RT Master Mix, 1 μg total RNA, and 9 μL nuclease-free water to reach a final volume. The reverse transcription conditions were as follows: incubation at 37 °C for 15 min to complete cDNA synthesis, heating at 85 °C for 5 s to terminate the reaction, and storage at 4 °C for subsequent use. RT-qPCR experiments were conducted using the TBGreen^®^ Premix Ex Taq™ II FAST qPCR Kit (Takara Bio Inc., Shiga, Japan) following strict adherence to standard operating procedures. Each 20 μL reaction system consisted of 10 μL TBGreen^®^ Premix Ex Taq™ II FAST qPCR Mix, 0.8 μL forward primer (10 μM), 0.8 μL reverse primer (10 μM), 2 μL cDNA template and 6.4 μL nuclease-free water. The PCR amplification program included initial denaturation at 95 °C for 30 s, followed by 40 cycles of denaturation at 95 °C for 5 s and annealing/extension at 60 °C for 30 s. RT-qPCR primers were designed using Primer-BLAST (Bethesda, MD, USA) from the NCBI database (Table S4). Hprt1 (hypoxanthine phosphoribosyl transferase 1) was adopted as a reference gene for gene expression normalization using three independent replicates per sample. The specificity of qPCR products were evaluated based on the melt curve analysis, and the relative expression levels of DEGs were calculated using the 2^−ΔΔCT^ method. RT-q PCR analysis was performed using GraphPad Prism 9, and statistical analysis was based on three independent experiments. Multiple *t* tests (and nonparametric tests) were used to evaluate the differences between groups. When *p* < 0.05, defined as statistically significant.

**Transmission electron microscopy analysis:** The ultrastructure of OVB-organoids was analyzed by transmission electron microscopy (TEM) analysis on the 10th, 20th, and 30th day of culture. OVB-organoids were washed with 1 × PBS and fixed with 2.5% glutaraldehyde (30 min, 4 °C). The samples were stored at 4 °C for transportation. The ultrastructure of OVB-organoids was evaluated using a TEM (Hitachi, Japan).

## 3. Results

### 3.1. Differential Analysis of Developmental Trajectories and Regulatory Mechanisms of Organoids

**Figure 1:**
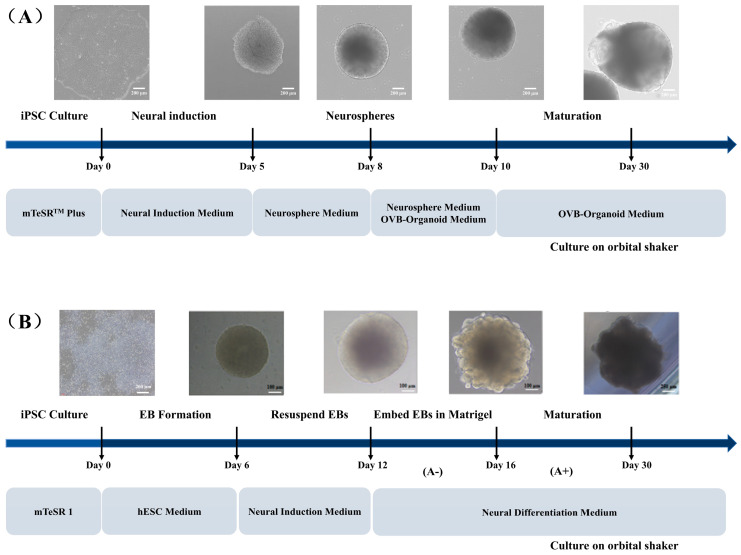
Protocol for the culture of cerebral organoids (**A**) and OVB-organoids (**B**) derived from iPSCs. (The transverse arrow represents the development process, and the vertical arrow represents the development node).

Through systematic time-series cultivation and morphological dynamic tracking (Figure 1), this study preliminarily revealed differences in the developmental dynamics and structural characteristics between cerebral organoids and OVB-organoids derived from hiPSCs. Although both share a common cellular origin, coordinated regulation by key growth factors and signaling pathway inhibitors guides hiPSCs toward specialization into different lineages.

In the medium used for cerebral organoid culture, Heparin (1 μg/mL) binds to and stabilizes growth factors such as FGF, thereby promoting the proliferation of neural precursor cells and the formation of neural vesicles [51,52,53]. In addition, ascorbic acid (vitamin C, 1% (*v*/*v*)) provides antioxidant protection and enhances neuronal maturation and synaptic function [54]. In contrast, the core regulatory strategy for OVB-organoid culture relies on dual signal blocking of SB431542 (a TGF-β inhibitor, 2.5–5 μM) and Dorsomorphin (a BMP inhibitor, 0.5 μM). Specifically, SB431542 suppresses the TGF-β/Smad pathway to prevent mesenchymal differentiation and promote neuroectodermal fate [55,56], whereas Dorsomorphin inhibits BMP signaling to further lock in the retinal pigment epithelium and neural retina lineages [57,58].

Vitamin A, a key regulator of neural development, was used in the culture of both types of organoids. It activates the retinoic acid receptors (RAR/RXR) to regulate gene expression, thereby influencing the regionalization, differentiation, and morphogenesis of neural progenitor cells [59,60,61,62]. In organoid culture, omitting vitamin A at the early stage prevents premature regionalization [30,63], allowing the spontaneous formation of brain-region structures driven by endogenous signals such as FGF and WNT [64]. In contrast, the addition of vitamin A at later stages promotes further regionalization of specific brain regions (e.g., cortex, basal ganglia, and hippocampus) and the differentiation of neuronal subtypes (e.g., glutamatergic and GABAergic neurons) [65,66].

During the early stages of culture, both organoid types exhibited cell aggregation and initially formed 3D structures. In cerebral organoids, the transfer of neuroectodermal tissues into Matrigel droplets promotes the formation and expansion of neuroepithelial buds, which gradually develop into typical neural rosette structures. In contrast, OVB-organoids are cultured in suspension in a liquid environment containing Matrigel; this setup facilitates self-assembly in 3D space, leading to the formation of organoids with more complex structures.

Although both organoids were derived from hiPSCs, distinct medium regulatory mechanisms enabled them to adopt unique strategies and achieve specific goals in simulating different developmental processes. By comparing these two culture methods, we can gain deeper insights into the complex mechanisms underlying cell differentiation, tissue morphogenesis, and functional maturation during the development of different organs (e.g., the brain and retina). This not only enhances our understanding of the potential and limitations of organoid technology in simulating human organ development but also provides valuable references for future research on disease modeling and regenerative medicine.

### 3.2. Differential Analysis of Immunofluorescence Staining Profiles of Organoids

Using immunofluorescence staining for specific markers, we elucidated phenotypic differences in cellular composition and spatial distribution between hiPSC-derived cerebral organoids and OVB-organoids. This further validates the precise role of distinct regulatory strategies in guiding hiPSC lineage specification, laying a phenotypic foundation for investigating the functional specificity of these two organoid types. Two groups of Day 30 organoids were used as research subjects to characterize the developmental status of the organoids, and immunofluorescence staining was performed for key markers associated with cell proliferation, neural stem cell (NSC) maintenance, and neuronal differentiation.

**Figure 2:**
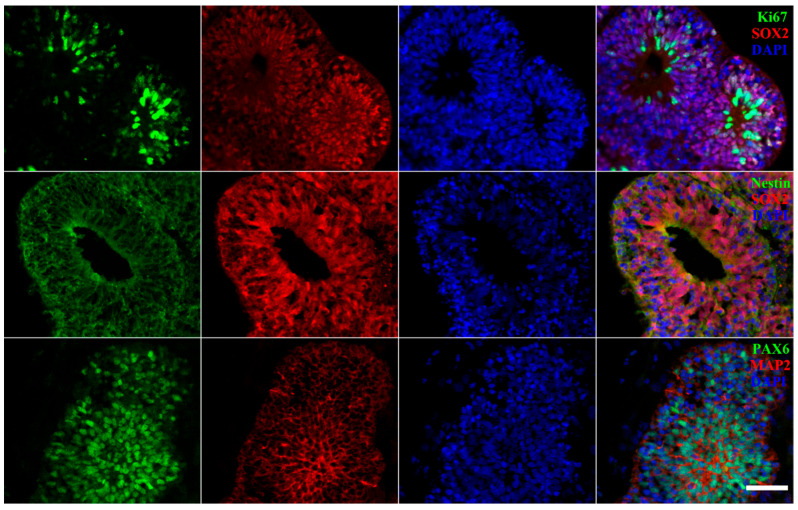
Co-localization of proliferation, neural stem cell, and differentiation markers in cerebral organoid: immunofluorescence staining of Ki67 (green), SOX2 (red), Nestin (green), PAX6 (green), and MAP2 (red) with nuclear counterstain (scale bar: 50 μm).

For cerebral organoids cultured up to Day 30, Ki67 and SOX2 co-staining was performed (Figure 2). This analysis revealed overlap between Ki67^+^ cells (a proliferation marker) and SOX2^+^ cells (a core NSC transcription factor), indicating that a substantial population of proliferating NSCs persists within the organoids. These cells constitute a sustained source for subsequent differentiation. Through Nestin/SOX2 co-staining of NSC markers, we observed significant co-expression of the two markers. In addition, the cells were arranged around the central cavity, forming a polar structure reminiscent of neuroepithelial or radial glial cells. This suggests that the system contained a rich population of NSCs and exhibited a polar neuroepithelial-like structure, aligning with the characteristics of NSC proliferation and maintenance. The staining analysis of the neuronal differentiation process revealed that PAX6 (a crucial factor in early neural differentiation and cortical development) was widely distributed, indicating the initiation of early neural fate determination and the development of cortical-like structures. Concurrently, the mature neuron marker MAP2 (red) exhibited specific staining, indicating that some cells had differentiated into mature neurons. Cerebral organoids are in a dynamic developmental stage that covers the process of NSC maintenance, directional differentiation, and early neuronal maturation. These cerebral organoids demonstrate a continuous developmental process, from NSC proliferation to neuronal maturation, showing the potential for further maturation into complex brain structures and functions.

**Figure 3:**
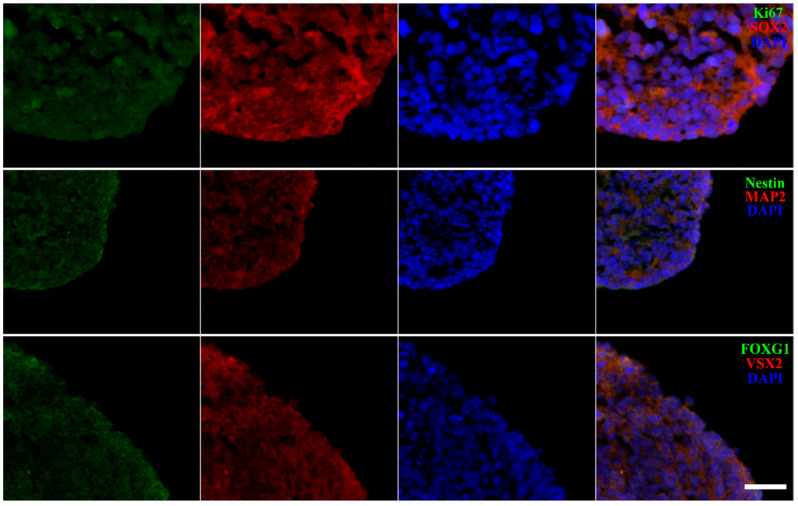
Co-localization of proliferation, neural progenitor, and regional specification markers in OVB-organoid: immunofluorescence staining of Ki67 (green), SOX2 (red), Nestin (green), MAP2 (red), FOXG1 (green), and VSX2 (red) with nuclear counterstain (Scale Bar: 50 μm).

The expression of key marker genes was detected using immunofluorescence staining to investigate the developmental characteristics of OVB-organoids cultured to Day 30 (Figure 3 and Figure S1). Ki67/SOX2 co-staining revealed widespread expression of both markers, indicating active cell proliferation, and that the cells either retained pluripotency or remained in the early stages of neural development. Nestin expression confirmed the presence of NSCs, whereas MAP2 expression indicated the initial formation of mature neurons. The combined analysis of these two markers suggests that the OVB-organoids are at the stage where NSCs differentiate into mature neurons. FOXG1, a key regulator of telencephalic development, exhibited fluorescent expression, demonstrating that telencephalon development-related programs were initiated. Furthermore, VSX2 (a critical marker gene for optic cup development) was expressed, indicating a developmental trend toward optic cup structure formation. Notably, the incomplete overlap of FOXG1 and VSX2 within OVB-organoids reflects the multistructural developmental properties of the system. The results indicate that OVB-organoids are currently in a transitional developmental stage, progressing from cell proliferation and pluripotency maintenance toward the differentiation of specific neural structures (such as the telencephalon and optic cup). Extensive Ki67 expression provides a sufficient cellular source to support organoid growth and subsequent differentiation. Sustained high expression of SOX2 lays a solid foundation for further differentiation of organoids into specific neural cell lineages. The expression of Nestin serves as direct evidence confirming the presence of NSCs within the organoids, whereas the weak expression of MAP2 indicates that the differentiation of cells into mature neurons has not yet been fully achieved. The concurrent expression of FOXG1 and VSX2 highlights the unique advantage of OVB-organoids for recapitulating the complexity of in vivo brain development. This property renders them an exceptional in vitro model for investigating the underlying molecular and cellular mechanisms governing coordinated development of the eye and brain. Furthermore, the OVB-organoid system is a powerful experimental approach for the comprehensive investigation of regulatory networks that orchestrate eye–brain developmental processes.

**Figure 4:**
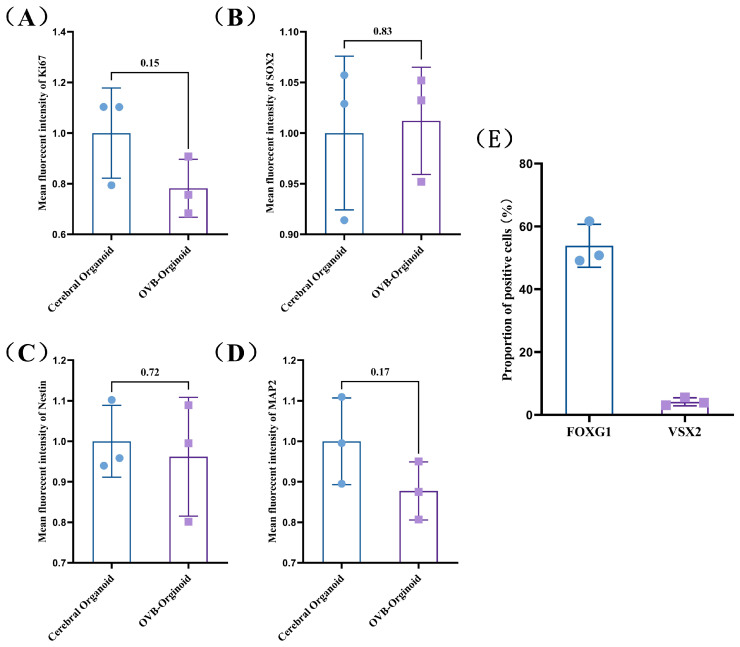
Semi-quantitative analysis of gene expression in cerebral organoid and OVB-organoid. (**A**–**D**) Mean fluorescent intensity of Ki67, SOX2, Notin, and MAP2 in cerebral organoid and OVB-organoid, analyzed by ImageJ. (**E**) Proportion of FOXG1^+^ and VSX2^+^ cells in the two organoids. (*n* = 3).

In this study, the semi-quantitative analysis of the immunofluorescence staining results was conducted for the two types of organoids using ImageJ software (Figure 4A–D). The results indicate that there are no significant differences in the expression levels of the four detected genes (Ki67, SOX2, Nestin and MAP2) between the two types of organoids, suggesting a high degree of similarity in gene expression patterns at this developmental stage, which indicates a relatively consistent developmental process. Additionally, the proportion of positive cells for FOXG1 and VSX2 was quantified, FOXG1^+^ cells accounted for 53.86% ± 5.58% in OVB-organoid, while VSX2^+^ cells accounted for 4.18% ± 1.04% (Figure 4E).

Collectively, the immunofluorescence staining results show that despite sharing a common cellular origin, these two types of organoids exhibited distinct developmental features and trajectories. Each type possesses a unique research value, offering complementary insights into neural development and related pathologies.

### 3.3. Differential Analysis of Transgenic Expression Profiles in Organoids

A comprehensive transcriptomic analysis of OVB-organoids and cerebral organoids was performed at the gene expression level to dissect potentially significant differences in developmental pathways, cellular composition, and functions between the two organoid types.

**Figure 5:**
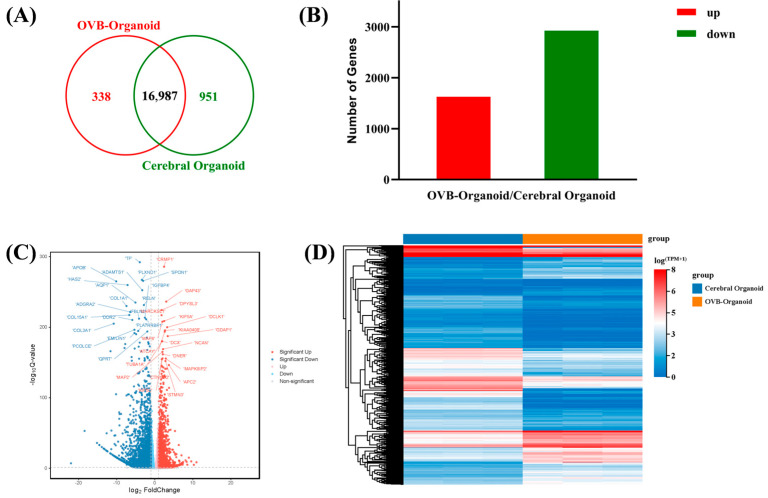
Transcriptomic analysis reveals DEGs between OVB-organoids and cerebral organoids. (**A**) Venn diagram of DEGs. (**B**) Histogram of DEGs. (**C**) Volcano plot of DEGs. (**D**) Heatmap of DEGs.

First, a Venn diagram (Figure 5A) visualized common and specific genes, which represented 16,987 co-expressed genes, whereas non-overlapping regions showed 338 and 951 specifically expressed genes in OVB-organoids and cerebral organoids, respectively. This identified similarities and differences in gene expression between the two organoids, reflecting potential disparities in their biological functions or developmental stages. Differential expression analysis was performed on all genes from both organoid types (|log2FC| >= 1 and Qvalue <= 0.05). A total of 1625 genes were up-regulated and 2924 genes were down-regulated in OVB-organoids relative to cerebral organoids (Figure 5B,C). This analysis not only demonstrates the statistical significance of gene expression changes but also visually illustrates their magnitude, providing crucial information for the subsequent identification of critical differential expressed genes. The expression patterns of differential genes were displayed using a heatmap (Figure 5D). Because these genes may participate in specific biological processes or signaling pathways, enrichment analysis was performed to assess the biological significance of the differential genes at a higher level.

**Figure 6:**
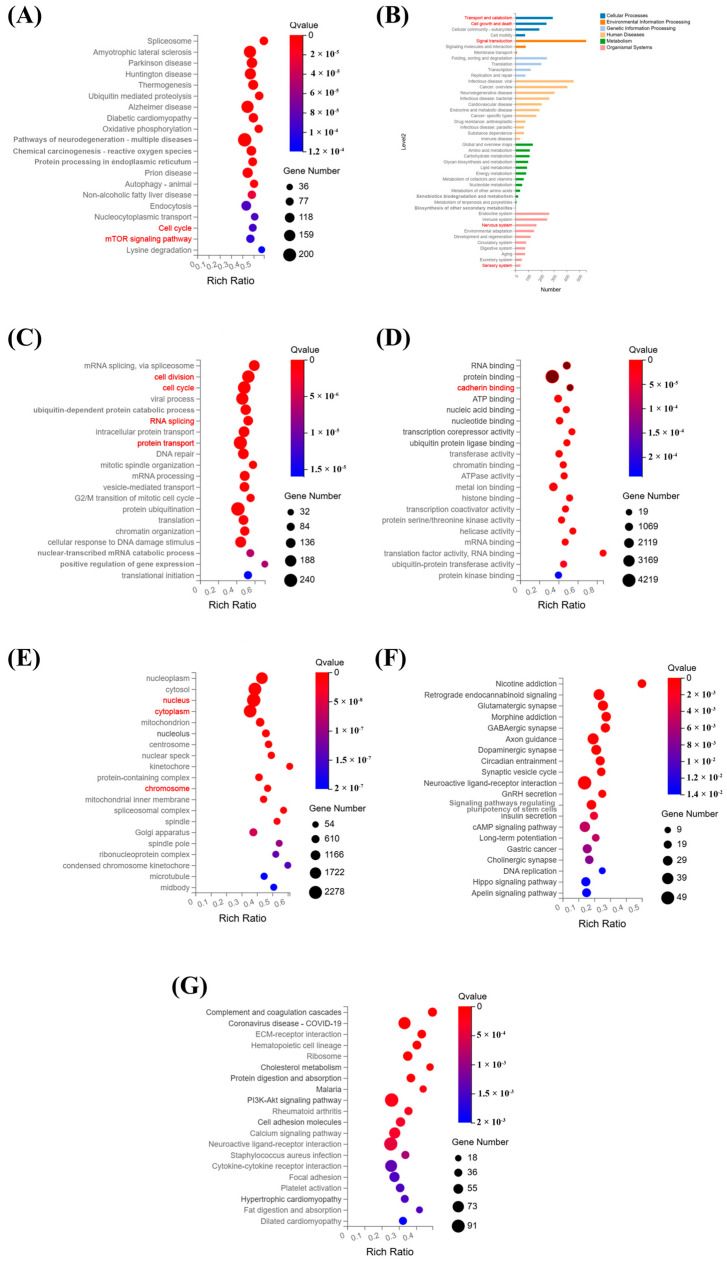
Transcriptomic analysis reveals functional and molecular differences between OVB-organoids and cerebral organoids. (**A**) KEGG pathway enrichment bubble chart. (**B**) Summary chart of KEGG pathway classifications. (**C**) GO biological process enrichment bubble chart. (**D**) GO molecular function enrichment bubble chart. (**E**) GO cellular component enrichment bubble chart. (**F**) KEGG pathway enrichment bubble chart of the up-regulated DEGs. (**G**) KEGG pathway enrichment bubble chart of the down-regulated DEGs. (The red channel is the channel that is focused on or mentioned).

KEGG enrichment analysis (Figure 6A) revealed that the DEGs were significantly enriched in pathways related to the cell cycle, proliferation, and signal transduction. These pathways are fundamental to organ morphogenesis and orderly cell growth, suggesting their potential involvement in the formation, development, and signal transduction of the optic cup. Further KEGG pathway classification (Figure 6B) showed significant enrichment of DEGs in cell growth and death pathways, as well as in transport and catabolism pathways within the cellular processes. These pathways play crucial roles in regulating cell growth, differentiation, and the formation and development of optic cup structures.

In the GO cellular component enrichment analysis (Figure 6C), DEGs were significantly enriched in pathways associated with cell division, cell cycle, RNA splicing, and protein transport. For the GO molecular function enrichment analysis (Figure 6D), differences in the brain region and optic cup development between the two organoids were reflected in the binding functions represented by cadherin binding. These functional pathways are involved in the regulation of critical molecular interactions, which may underlie the phenotypic differences observed during organoid development. In the GO biological-process enrichment analysis (Figure 6E), significant enrichment was detected in pathways, including the nucleus, cytoplasm, and chromosome. These biological processes are essential for the differentiation, development, and functional establishment of retinal cells and their associated structures. In Figure 6F,G, KEGG enrichment analysis was used to analyze the up-and down-regulated genes. Compared with cerebral organoids, the up-regulated genes in OVB-organoids were significantly enriched in multiple neural development and synaptic function pathways (such as glutamatergic synapse, GABA synapse, and long-term potentiation). In contrast, down-regulated genes were mainly enriched in immune inflammatory pathways (such as complement and coagulation cascades) and metabolic/adhesion pathways (such as cholesterol metabolism, ECM–receptor interaction). The specific information of the pathway is shown in Tables S6 and S7.

**Figure 7:**
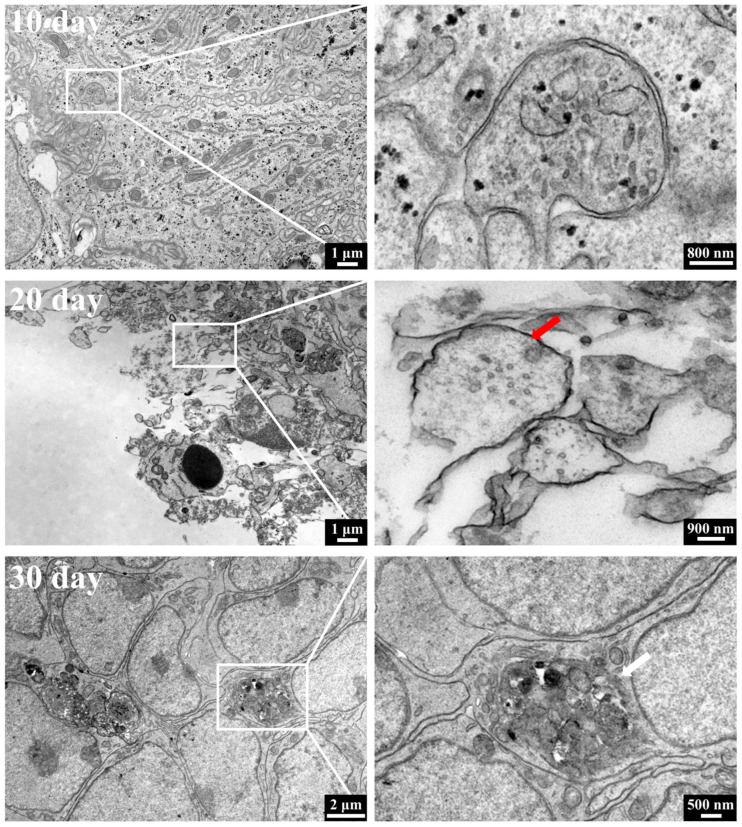
TEM characterization of ultrastructure of OVB-organoids.(The red arrow is synapses and synaptic vesicles, and the white arrow is myelin-like structures).

As shown in Figure 7, a small number of synapses and synaptic vesicles (indicated by red arrows) have appeared in the OVB-organoids on day 20. The pre- and post-synaptic membrane structures are dense and well-defined, with synaptic vesicles aggregating in a circular or oval shape in the pre-synaptic membrane region. This indicates that the neurons have matured in development, and functional synaptic connections have begun to form between neurons, suggesting that the neural network of the constructed brain organoids has entered a stage of functional maturation. In the OVB-organoids on day 30, myelin-like structures (indicated by white arrows) can be observed, suggesting that glial cell differentiation and maturation have occurred within the organoids, and the formation of myelin sheaths around the neural axons has begun. The appearance of myelin structures indicates that these OVB-organoids possess a favorable neurodevelopmental microenvironment, capable of supporting the synchronous processes of neuronal maturation, synapse formation, and myelination, thus enabling the coordinated development of neurons and glial cells, providing an important morphological basis for the validation of neural functions.

**Figure 8:**
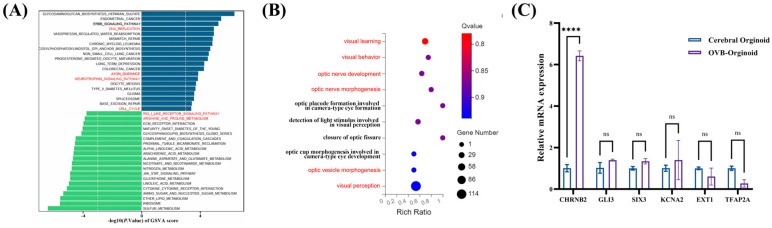
(**A**) GSVA pathway enrichment chart. (**B**) Enrichment bubble chart of biological processes related to visual development. (**C**) Relative mRNA expression bar chart for comparing the expression levels of visual-related genes. The marker red pathway is the pathway that is focused on or mentioned. The statistical data were expressed as Mean ± SD, and the number of samples was *n* = 3. Using multiple *t* test analysis, the significance level was expressed as **** *p* < 0.0001.

Gene set enrichment analysis (Figure 8A) demonstrated that OVB-organoids exhibited stronger activity in DNA replication, neurotrophin signaling pathway and cell cycle, indicating that OVB-organoids were more active in biosynthesis and cell proliferation. In contrast, cerebral organoids showed high activity in metabolism-related pathways (for example, glycine, serine and threonine metabolism) and signal transduction pathways (for example, RIG-I-like receptor signaling pathway).

Visual pathway enrichment analysis (Figure 8B) revealed pathway activation related to optic cup formation, optic-nerve-structure formation, and visual learning in the OVB-organoids. This suggests that OVB-organoids may have the potential to develop into visual-related neural structures (such as optic cup, optic nerve).

Six visual-related DEGs were selected for verification via RT-qPCR (Figure 8C). Most of these genes exhibited expression patterns consistent with the transcriptomic data.

In summary, OVB-organoids exhibit significant differences from cerebral organoids in simulating early retinal development, constructing optic cup and optic nerve structures, forming visual-related neural circuits, and achieving visual functions. These characteristics render OVB-organoids an important experimental model for research into visual diseases and regenerative medicine.

## 4. Discussion

This study systematically compared the differences between OVB-organoids and cerebral organoids cultured for 30 days in terms of culture protocols, cellular composition, molecular characteristics, and functional simulation, providing important data for the optimization and application of organoid models. In terms of culture protocols, OVB-organoids exhibit more distinct regionalized structural features, whereas cerebral organoids, although capable of generating diverse types of neural cells, show higher randomness in regional distribution. Immunofluorescence staining tests further confirm that the developmental programs for the brain and optic cup occurred simultaneously during the co-expression of FOXG1 and VSX2 in OVB-organoids, reflecting specific multistructural developmental characteristics. Transcriptome analysis reveals fundamental differences between the two organoid models: OVB-organoids display more significant co-expression of eye–brain gene modules, including differential activation of specific genes, such as CHRNB2 and SIX3. RT-qPCR validation further highlighted the gradient differences in the expression of vision-related markers between the two types of organoids, consistent with the developmental trends revealed by single-cell sequencing. At the same time, the formation of synapses and myelin-like structures in OVB-organoids was verified by TEM analysis.

From a technical application perspective, OVB-organoids provide a more precise in vitro model for studying early development of the eye and brain, with their inherent capacity for spontaneous formation effectively avoiding the operational complexity associated with artificial fusion—an advantage conventional cerebral organoids lack. Critically, in disease modeling, OVB-organoids uniquely recapitulate pathogenic mechanisms of eye–brain interaction disorders, acting as a next-generation organoid to simulate retinal lesions from early neurodevelopmental defects. Although cerebral organoids remain irreplaceable for global whole-brain or widespread neurodevelopmental disorder modeling (for example, microcephaly and autism), OVB-organoids fill the key gap in simulating visual–brain tissue-specific interactions, providing a targeted translational platform.

Despite the promising developments of this study, it is still in its early stages and has limitations. First, OVB-organoids encounter technical challenges in long-term culture, which restricts their maturation. Second, although a standardized construction protocol has been established, variability between batches persists. Third, the absence of functional validation, such as electrophysiological assessments, hinders a thorough evaluation of the functionality of OVB-organoids.

The key findings of this study highlight the unparalleled advantages of OVB-organoids in simulating eye–brain integrated development and related disease research, offering empirical support for model selection. Future efforts to optimize the long-term culture, minimize differentiation, and integrate functional assays will enhance the utility of OVB-organoids as a transformative tool in neuroscience.

## 5. Patents

A patent resulted from the work reported in this manuscript, and its title is A Human-Induced Pluripotent Stem Cell-Derived Optic Vesicle-Containing Brain Organoid and Method.

## Data Availability

The authors confirm that the data supporting the findings of this study are available within the article and its Supplementary Materials.

## Supplementary Materials

The following supporting information can be downloaded at: https://www.mdpi.com/article/10.3390/cells15080703/s1, Figure S1: Co-localization of Proliferation, Neural Progenitor, and Regional Specification Markers in OVB-Organoid: Immunofluorescence Staining of Ki67, SOX2, Nestin, MAP2, FOXG1, and VSX2 with Nuclear Counterstain (Scale Bar: 50 µm); Table S1: Reagents and Materials for iPSC and Organoid Culture and Characterization; Table S2: Composition of Neurosphere Medium; Table S3: Composition of OVB-organoid Medium; Table S4: RT-qPCR primer sequences; Table S5: The TOP 20 DEGs between OVB-Organoids and Cerebral Organoids; Table S6: Genes significantly down-regulated in Cerebral Organoids compared to OVB-organoids and their related enrichment pathway information; Table S7: Genes significantly up-regulated in Cerebral Organoids compared to OVB-organoids and their related enrichment pathway information.

## Abbreviations

The following abbreviations are used in this manuscript:

OVB-organoid	optic vesicle-containing brain organoid
hiPSC	human-induced pluripotent stem cell
iPSC	induced pluripotent stem cell
3D	three-dimensional
PSCs	pluripotent stem cells
NMOs	neuromuscular organoids
KEGG	Kyoto Encyclopedia of Genes and Genomes
GO	Gene Ontology
EDTA	Ethylene Diamine Tetraacetic Acid
DPBS	Dulbecco’s Phosphate Buffered Saline
bFGF	basic Fibroblast Growth Factor
EBs	Embryoid Bodies
NIM	Neural Induction Medium
PFA	Paraformaldehyde
RT	Room Temperature
O.C.T.	Optimal Cutting Temperature compound
PBST	PBS with Tween-20
DAPI	4′,6-Diamidino-2-Phenylindole
DEG	Differentially Expressed Gene
RT-qPCR	Real-time quantitative polymerase chain reaction
FGF	Fibroblast Growth Factor
TGF-β	Transforming Growth Factor-β
BMP	Bone Morphogenetic Protein
RAR	Retinoic Acid Receptor
RXR	Retinoid X Receptor
WNT	Wingless/Integrated
NSC	Neural Stem Cell
FC	Fold Change
GSVA	Gene Set Variation Analysis
TEM	Transmission Electron Microscope
CHRNB2	Cholinergic Receptor Nicotinic Beta 2 Subunit
SIX3	Sine Oculis Homeobox 3
ROCK	Rho-associated coiled-coil containing protein kinase
MAP2	Microtubule-associated protein 2
TBGreen	Transcription-Buffered Green
Hprt1	Hypoxanthine Phosphoribosyl Transferase 1
SOX2	SRY-Box Transcription Factor 2
PAX6	Paired Box 6
Ki67	Ki-67 Antigen
TBR2	T-Box Brain 2
FOXG1	Forkhead Box G1
VSX2	Visual System Homeobox 2
