# Supplementary material for: Multiscale Comparative Analyses of OVB-Organoids and Cerebral Organoids
Source: Cells. 2026 Apr 16;15(8):703. doi: 10.3390/cells15080703 (PMC13114402; doi:10.3390/cells15080703)
Supplement: Supplementary file 1 [file cells-15-00703-s001.zip › cells-4182492-supplementary.pdf]

Figure legends

Figure S1. Co-localization of Proliferation, Neural Progenitor, and Regional Specifica tion Markers in OVB-Organoid: Immunofluorescence Staining of Ki67, SOX2, Nestin, MAP2, FOXG1, and VSX2 with Nuclear Counterstain (Scale Bar: 50 μm)

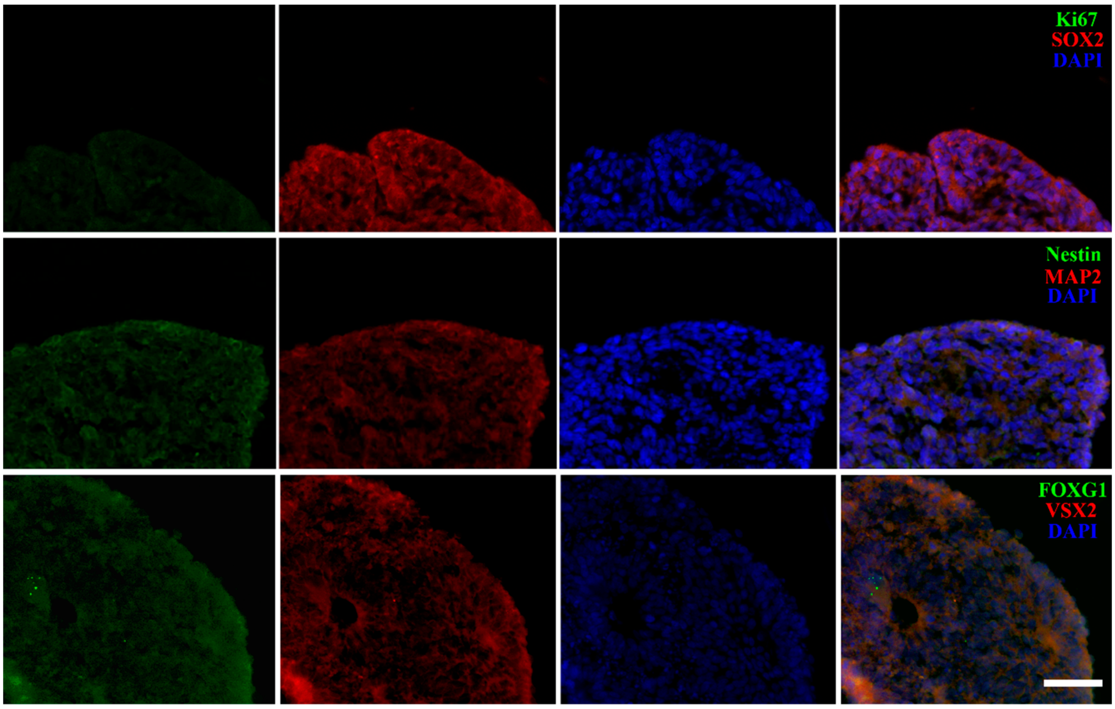

Figure S1. Co-localization of Proliferation, Neural Progenitor, and Regional Specification Markers in OVB-Organoid: Immunofluorescence Staining of Ki67, SOX2, Nestin, MAP2, FOXG1, and VSX2 with Nuclear Counterstain (Scale Bar: 50 μm)

Tables legends

Table S1. Reagents and Materials for iPSC and Organoid Culture and Characterization.

Table S2. Composition of Neurosphere Medium

Table S3. Composition of OVB-organoid Medium

Table S4. RT-qPCR primer sequences

Table S5. The TOP 20 DEGs between OVB-Organoids and Cerebral Organoids

Table S6. Genes significantly down-regulated in Cerebral Organoids compared to OVB-organoids and their related enrichment pathway information

Table S7. Genes significantly up-regulated in Cerebral Organoids compared to OVB-organoids and their related enrichment pathway information

Table S1. Reagents and Materials for iPSC and Organoid Culture and Characterization.

Reagent or Resource	Source	Identifier
	Cell line	
iPSC	CELLAPY	CA4039106
	Antibodies	
Anti-SOX2	Abcam	Cat#: ab97959
Anti-Ki67	Abcam	Cat#: ab15580
Anti-FOXG1	Abcam	Cat# ab196868
Anti-MAP2	Abcam	Cat#: ab32454
Anti-Nestin	Abcam	Cat# ab313787
Anti-PAX6	Abcam	Cat# ab195045
Anti-VSX2	Proteintech	Cat# 25825-1-AP
Alexa Fluor 488 -goat anti- rabbit lgG (H+L)	Life technologies	Cat# ab150077
Alexa Fluor 594 -Goat anti-mouse IgG (H+L)	abcam	Cat# ab150116
DAPI	Sigma-Aldrich	32670
	Chemicals, peptides, recombinant proteins, and other reagents	
DMEM/F12	Thermo Scientific	10565018
mTeSR^{TM} Plus	Stem cell technologies	#05825
mTeSR1	Stem cell technologies	#05850
Matrigel hESC-qualified matrix	Corning	354277
Rock-inhibitor (Y-27632 2HCI)	Selleck	S1049
PBS(1×)	Solarbio	P1020-500ML
PBS (10×)	Solarbio	P1022-500ML
PBST(10×)	Solarbio	P1033-500ML
ReLeSR^{TM}	Stem cell technologies	# 05872
Accutase solution	Sigma-Aldrich	A6964-100ML
Neural induction medium (NIM)	Stem cell technologies	05835
Neural basal medium	Thermo Scientific	21103049
N2 supplement	Thermo Scientific	17502048
B-27 supplement	Thermo Scientific	17504044

B-27supplement w/o Vitamin A	Thermo Scientific	12587010
Glutamax	Thermo Scientific	35050061
MEM	Thermo Scientific	11140050
Insulin	Sigma-Aldrich	13536-100MG
SB431542	Selleck	S1067
Penicillin/Streptomycin	Thermo Scientific	15140122
Beta-Mercaptoethanol	Thermo Scientific	31350010
Dorsomorphin	Sigma-Aldrich	P5499
EDTA	Sigma-Aldrich	E6758
bFGF	Peprotech	100-18B
Heparin	Sigma	H3149
Chromium Single Cell 3 Reagent Kits v3	TIANGEN	DP419
DirectZol RNA kit	TIANGEN	KR118-02
Fluoromount-G®	SouthernBiotech	0100-01
Citrate Antigen Retrieval Solution（50×）	Solarbio	C1032
Super PAP Pen	ZSGB-BIO	ZLI-9305
Tissue-Tek O.C.T. Compound	Sakura	4583
Paraformaldehyde,4%	Solarbio	P1110
Triton-X100	Beyotime	P0096-100ml
Goat Serum	Beyotime	C0265

Table S2. Composition of Neurosphere Medium

Neurosphere medium	
component	Final conc.
DMEM/F12	48.40% (v/v)
Neural basal medium	48.40% (v/v)
N2	0.4X
B27 w/o Vitamin A	0.2X
Glutamax	1X
MEM	0.5X
Insulin	0.2755 μM
SB431542	2.5 μM
Pen/Strep	100 U/mL
Beta-Mercaptoethanol	5 μM
Matrigel	0.1%

Table S3. Composition of OVB-organoid Medium

OVB-organoid medium	
component	Final conc.
DMEM/F12	48.40% (v/v)
Neural basal medium	48.40% (v/v)
N2	0.4X
B27 + Vitamin A	0.2X
Glutamax	1X
MEM	0.5X
Insulin	0.2755 μM
SB431542	2.5 μM
Pen/Strep	100 U/mL
Beta-Mercaptoethanol	5 μM
Dorsomorphin	0.5 μM

Table S4. RT-qPCR primer sequences

Gene	Primer sequences
CHRNB2	GAAAGTTCGGCTCCCTTCCA
	GCTGGTCAAATGGGAAGTGC
GLI3	TTCGGGGACTTGACAGCCG
	GGACTGGGCCTCCATGATGT
SIX3	GCTTCTCCTTTCGGTTCCCA
	TGTCCTCGTGGTGGTGATTG
KCNA2	CCGAGGAGATGTGAGGGATTT
	TTGAAGACAGCCGAGCATGG
EXT1	GCATGGCAAAGACTGGCAAA
	GACCACGAGGAACCAGACAG
TFAP2A	GAGTAGCTCCACTTGGGTGC
	CCTCGCAGTCCTCGTACTTG

Table S5. The TOP 20 DEGs between OVB-Organoids and Cerebral Organoids

Gene ID	Gene Symbol	log2FoldChange	q-value	Average TPM of OVB-Organoid	Average TPM of Cerebral Organoid	Trend
7018	TF	-3.96119	7.71E-293	1.33	19.68	down
23129	PLXND1	-3.41446	7.79E-268	4.39	45.49	down
10418	SPON1	-3.07712	2.29E-266	6.63	51.94	down
338	APOB	-10.1616	1.02E-265	0.06	70.08	down
3037	HAS2	-7.16968	1.64E-260	0.51	67.32	down
9510	ADAMTS1	-3.29982	3.57E-253	5.16	47.30	down
358	AQP1	-5.10312	1.09E-235	1.94	61.34	down
5649	RELN	-2.88029	3.76E-232	7.21	49.76	down
1277	COL1A1	-7.45264	9.99E-231	3.19	518.56	down
25960	ADGRA2	-6.47638	2.81E-222	0.33	27.38	down
1306	COL15A1	-6.71906	5.28E-218	0.39	38.13	down
3487	IGFBP4	-2.6093	3.55E-214	28.44	157.48	down
2199	FBLN2	-4.14889	7.76E-213	3.01	49.58	down
6238	RRBP1	-2.27802	6.22E-212	19.70	89.62	down
4921	DDR2	-5.91584	5.03E-211	0.29	16.41	down
1281	COL3A1	-10.8167	9.54E-206	1.24	2092.27	down
5118	PCOLCE	-5.36234	4.25E-197	3.05	111.63	down
5327	PLAT	-4.38358	2.3E-195	2.73	52.03	down
23475	QPRT	-1.96746	1.29E-194	78.80	281.84	down
11117	EMILIN1	-5.34832	1.98E-192	0.90	34.10	down
1400	CRMP1	2.43612	1.67E-286	371.35	63.96	up
2596	GAP43	2.9951	5.46E-237	229.12	25.55	up
1809	DPYSL3	1.7793	5.44E-225	294.84	79.69	up
65108	MARCKSL1	1.75804	9.78E-218	1347.28	355.03	up

3798	KIF5A	2.46078	1.74E-209	53.21	9.08	up
4135	MAP6	1.98293	1.46E-208	178.39	38.02	up
9201	DCLK1	3.23816	1.21E-200	40.19	3.72	up
9729	KIAA0408	2.65947	3.48E-196	37.78	5.6	up
85300	ATCAY	2.60223	2.05E-194	58.46	8.87	up
54332	GDAP1	3.38758	5.16E-188	67.17	6.17	up
1641	DCX	1.92589	8.75E-181	94.64	23.39	up
7846	TUBA1A	1.88711	1.18E-180	5125.99	1240.93	up
1463	NCAN	2.15734	1.23E-169	46.67	9.76	up
4133	MAP2	1.99271	9.19E-166	189.33	45.02	up
92737	DNER	2.09988	2.1E-162	146.13	31.42	up
1501	CTNND2	2.02654	1.64E-156	75.40	16.79	up
23542	MAPK8IP2	2.88077	2.56E-156	27.49	3.48	up
3800	KIF5C	1.82107	2.72E-155	121.99	32.45	up
50861	STMN3	2.79724	8.11E-153	95.94	12.55	up
10297	APC2	3.69653	4.03E-147	33.44	2.41	up

Table S6. Genes significantly down-regulated in Cerebral Organoids compared to OVB-organoids and their related enrichment pathway information

KEGG Pathway Term ID	KEGG Pathway Term Desc	KEGG Pathway Term Level1	KEGG Pathway Term Level2	Term Candidate Gene Num	Term Gene Num	Rich Ratio	P value	Q value	Gene Symbols
5033	Nicotine addiction	Human Diseases	Substance dependence	20	40	0.5	3.56E-13	1.14E-10	CHRNB2,GRIN3A,SLC32A1,SLC17A8,GABRA1,GABRA3,GABRB2,GABRB3,GABRD,GABRG1,GABRG2,GRIA1,GRIA2,GRIA3,GRIA4,GRIN1,GRIN2B,SLC17A6,CACNA1A,CACNA1B
4723	Retrograde endocannabinoid signaling	Organismal Systems	Nervous system	34	148	0.22972973	7.54E-10	1.17E-07	MGLL,ADCY8,CNR1,SLC32A1,RIMS1,SLC17A8,GABRA1,GABRA3,GABRB2,GABRB3,GABRD,GABRG1,GABRG2,GNAO1,GNG3,GNG4,GRIA1,GRIA2,GRIA3,GRIA4,GRM1,GRM5,KCNJ5,KCNJ6,KCNJ9,GNG2,PRKACB,PRKCB,MAPK11,MAPK10,SLC17A6,CACNA1A,CACNA1B,GNG8
4724	Glutamatergic synapse	Organismal Systems	Nervous system	29	114	0.254385965	1.10E-09	1.17E-07	ADCY8,GRIN3A,SLC17A8,GNAO1,GNG3,GNG4,GRIA1,GRIA2,GRIA3,GRIA4,GRIK1,GRIK2,GRIK3,GRIN1,GRIN2B,GRM1,GRM2,GRM3,GRM5,GRM7,GRM8,SHANK1,GNG2,PRKACB,PRKCB,SLC17A6,SLC1A2,CACNA1A,GNG8

5032	Morphine addiction	Human Diseases	Substance dependence	25	91	0.274725275	2.81E-09	2.25E-07	ADCY8,SLC32A1,GABRA1,GABRA3,GABRB2,GABRB3,GABRD,GABRG1,GABRG2,GNAO1,GNG3,GNG4,KCNJ5,KCNJ6,KCNJ9,PDE1A,PDE2A,PDE4B,GNG2,PRKACB,PRKCB,CACNA1A,CACNA1B,GNG8,GABBR2
4727	GABAergic synapse	Organismal Systems	Nervous system	24	89	0.269662921	8.79E-09	5.62E-07	ADCY8,SLC32A1,GABRA1,GABRA3,GABRB2,GABRB3,GABRD,GABRG1,GABRG2,GAD2,GNAO1,GNG3,GNG4,KCNJ6,PLCL1,GNG2,PRKACB,PRKCB,SLC12A5,SLC6A1,CACNA1A,CACNA1B,GNG8,GABBR2
4360	Axon guidance	Organismal Systems	Development and regeneration	35	182	0.192307692	5.82E-08	3.10E-06	PLXNC1,SEMA6C,CFL1,UNC5D,DCC,DPYSL2,EFNA3,EFNA5,EPHA3,EPHA4,EPHA7,EPHB1,SEMA3D,NTNG1,RND1,HRAS,L1CAM,PAK3,PARD6A,PLXNA1,SEMA5B,DPYSL5,RGMA,PAK5,NTN4,LRRC4,BMPR1B,TRPC4,TRPC5,CXCR4,CAMK2B,ABLIM2,PIK3R3,SEMA3E,SRGAP3
4728	Dopaminergic synapse	Organismal Systems	Nervous system	28	132	0.212121212	1.51E-07	6.88E-06	ATF6B,DRD4,GNAO1,GNG3,GNG4,GRIA1,GRIA2,GRIA3,GRIA4,GRIN2B,KCNJ5,KCNJ6,KCNJ9,KIF5A,KIF5C,BMAL1,GNG2,PPP2R2B,PPP2R2C,PRKACB,PRKCB,MAPK11,MAPK10,SCN1A,CACNA1A,CACNA1B,CAMK2B,GNG8
4713	Circadian entrainment	Organismal Systems	Environmental	23	97	0.237113402	2.39E-07	9.57E-06	ADCY8,ADCYAP1,GNAO1,GNG3,GNG4,GRIA1,GRIA2,GRIA3,GRIA4,GRIN1,GRIN2B,GUCY1A2,KCNJ5,KCNJ6,KCNJ9,GNG2,PR

			adaptation						KACB,PRKCB,RYR2,RYR3,CAMK2B,GNG8,NOS1AP
4721	Synaptic vesicle cycle	Organismal Systems	Nervous system	19	78	0.243589744	1.73E-06	6.16E-05	CPLX2,STX1B,SLC32A1,ATP6V0E2,RIMS1,UNC13A,SLC17A8,DNM3,ATP6V0C,ATP6V1G2,SLC17A6,RAB3A,SLC1A2,SLC6A1,SNAP25,SYT1,CACNA1A,CACNA1B,ATP6V0D1
4080	Neuroactive ligand-receptor interaction	Environmental Information Processing	Signaling molecules and interaction	49	353	0.138810198	5.10E-06	1.63E-04	HRH3,CHRM4,CHRNB2,ADCYAP1,GRIN3A,CNR1,CRHR1,ADRA1B,ADRA2A,DRD4,GABRA1,GABRA3,GABRB2,GABRB3,GABRD,GABRG1,GABRG2,GLP1R,GLRA2,GRIA1,GRIA2,GRIA3,GRIA4,GRID1,GRID2,GRIK1,GRIK2,GRIK3,GRIN1,GRIN2B,GRM1,GRM2,GRM3,GRM5,GRM7,GRM8,HCRTR2,HTR1A,OPRK1,PENK,PTGER3,TAC1,TACR3,GLRA3,KISS1R,NPFF,CCKAR,GPR50,GABBR2
4929	GnRH secretion	Organismal Systems	Endocrine system	16	64	0.25	7.95E-06	2.31E-04	HRAS,HCN1,KCNJ5,KCNJ6,KCNJ9,KCNN1,KCNN3,PRKCB,HCN3,HCN2,SPP1,TRPC4,TRPC5,KISS1R,PIK3R3,GABBR2
4550	Signaling pathways regulating pluripotency of stem cells	Cellular Processes	Cellular community - eukaryotes	26	143	0.181818182	8.81E-06	2.35E-04	APC2,FZD10,DLX5,ONECUT1,APC,HRAS,ID4,NEUROG1,OTX1,PAX6,MAPK11,SMARCAD1,LHX5,BMI1,BMPR1B,WNT1,WNT6,WNT7A,WNT8B,WNT2B,WNT9B,ZIC3,WNT10A,FZD1,PIK3R3,KLF4

4911	Insulin secretion	Organismal Systems	Endocrine system	17	86	0.197674419	1.09E-04	0.002672298	KCNMB2,ADCY8,ADCYAP1,ATF6B,GLP1R,KCNN1,KCNN3,ATP1A3,PRKACB,PRKCB,RAB3A,RYR2,SNAP25,ABCC8,CAMK2B,CCKAR,RIMS2
4024	cAMP signaling pathway	Environmental Information Processing	Signal transduction	31	221	0.140271493	2.36E-04	0.005040941	HCN4,ADCY8,ADCYAP1,GRIN3A,CNGB1,CNGA3,CNGA4,CRHR1,GLP1R,GRIA1,GRIA2,GRIA3,GRIA4,GRIN1,GRIN2B,HTR1A,LIPE,ATP1A3,ATP2B2,PDE4B,PRKACB,MAPK10,PTGER3,HCN2,RYR2,BDNF,SOX9,CAMK4,CAMK2B,PIK3R3,GABBR2
4720	Long-term potentiation	Organismal Systems	Nervous system	14	67	0.208955224	2.36E-04	0.005040941	ADCY8,GRIA1,GRIA2,GRIN1,GRIN2B,GRM1,GRM5,HRAS,PPP1R1A,PRKACB,PRKCB,RPS6KA2,CAMK4,CAMK2B
5226	Gastric cancer	Human Diseases	Cancer: specific types	23	148	0.155405405	3.42E-04	0.006843404	FRAT1,APC2,CDKN2B,FZD10,CTNNA2,E2F2,APC,HRAS,MLH1,ABCB1,SHC3,TGFBR1,WNT1,WNT6,WNT7A,WNT8B,WNT2B,WNT9B,WNT10A,FZD1,PIK3R3,FGF17,CCNE2
4725	Cholinergic synapse	Organismal Systems	Nervous system	19	113	0.168141593	4.04E-04	0.007599174	CHRM4,ADCY8,CHRNB2,GNAO1,GNG3,GNG4,HRAS,KCNJ6,KCNQ2,KCNQ3,GNG2,PRKACB,PRKCB,CACNA1A,CACNA1B,CAMK4,CAMK2B,PIK3R3,GNG8
3030	DNA replication	Genetic Information	Replication and repair	9	36	0.25	7.87E-04	0.01353794	DNA2,FEN1,POLA2,MCM2,MCM5,MCM7,PCNA,POLE,POLE2

		Processing							
4390	Hippo signaling pathway	Environmental Information Processing	Signal transduction	23	157	0.146496815	8.04E-04	0.01353794	APC2,FZD10,CCN2,CTNNA2,DLG2,GLI2,APC,PARD6A,PPP2R2B,PPP2R2C,BMPR1B,TGFBR1,TP73,WNT1,WNT6,WNT7A,WNT8B,WNT2B,WNT9B,WNT10A,GDF5,FZD1,TEAD2
4371	Apelin signaling pathway	Environmental Information Processing	Signal transduction	21	139	0.151079137	8.94E-04	0.01384061	PPARGC1A,ADCY8,CCN2,GNG3,GNG4,HRAS,LIPE,MYLK,NOS2,GNG2,PRKACB,SPHK2,RYR2,RYR3,SLC8A2,SLC8A1,SLC8A3,SPP1,TGFBR1,CAMK4,GNG8

Table S7. Genes significantly up-regulated in Cerebral Organoids compared to OVB-organoids and their related enrichment pathway information

KEGG Pathway Term ID	KEGG Pathway Term Desc	KEGG Pathway Term Level1	KEGG Pathway Term Level2	Term Candidate Gene Num	Term Gene Num	Rich Ratio	P value	Q value	Gene Symbols
4610	Complement and coagulation cascades	Organismal Systems	Immune system	42	85	0.494118	2.06E-12	7.23E-10	PROCR,CPB2,CR1L,A2M,F2,F2RL2,F3,F7,F10,F12,FGA,FGB,FGG,SERPIND1,CFI,ITGAM,ITGAX,ITGB2,KNG1,SERPINC1,SERPINE1,SERPINA5,SERPINA1,PLAT,PLAU,PLG,SERPINF2,PROC,PROS1,BDKRB1,BDKRB2,CFB,TFPI,THBD,C1R,C1S,C2,C3,C4B,C5AR1,VTN,VWF
5171	Coronavirus disease - COVID-19	Human Diseases	Infectious disease: viral	77	232	0.331897	2.59E-10	4.55E-08	RPL36A-HNRNPH2,RPS10-NUDT3,IRF9,RPL35,RPL10L,RPL22L1,F2,FCGR2A,FGA,FGB,FGG,FOS,RPL36,STING1,IL6R,IL6ST,MMP1,MX1,MX2,MYD88,RPS27L,RPL3L,RPL9,RPL10,RPL11,RPL12,RPL17,RPL18,RPL23A,RPL24,RPL27,RPL30,RPL27A,RPL28,RPL29,RPL31,RPL32,RPL34,RPL35A,RPL36AL,RPL37,RPL37A,RPL38,RPL39,RPL36A,RPLP2,RPS3A,RPS8,RPS12,RPS13,RPS14,RPS15A,RPS17,RPS18,RPS21,RPS24,RPS25,RPS27,RPS27A,RPS28,RPS29,CFB,CCL2,SELP,IFIH1,TLR2,

									TLR4,C1R,C1S,C2,C3,C4B,C5AR1,VWF,NRP1,ISG15,IKBKE
4512	ECM-receptor interaction	Environmental Information Processing	Signaling molecules and interaction	38	88	0.431818	3.19E-09	3.74E-07	LAMC3,CHAD,COL1A1,COL1A2,COL4A1,COL4A2,COL6A1,COL6A2,COL6A3,COL9A3,COMP,FREM1,ITGA11,FN1,NPNT,GP1BA,GP9,HSPG2,TNC,FREM2,ITGA1,ITGA2B,ITGA5,ITGA9,ITGB3,LAMA2,LAMA5,LAMB1,LAMB3,LAMC1,RELN,SDC4,TNXB,VTN,VWF,ITGA10,ITGA8,CD44
4640	Hematopoietic cell lineage	Organismal Systems	Immune system	40	99	0.40404	1.26E-08	1.11E-06	CR1L,CSF1,CSF1R,CSF2RA,EPOR,FLT3LG,GP1BA,GP9,ANPEP,GYPA,HLA-DMA,HLA-DMB,HLA-DOA,HLA-DOB,HLA-DPA1,HLA-DPB1,HLA-DQB1,HLA-DRB1,HLA-DRB3,IL1R1,IL3RA,IL4R,IL6R,IL9R,IL11,IL11RA,ITGA1,ITGA2B,ITGA5,ITGAM,ITGB3,MME,THPO,CD1D,CD4,CD7,CD8B,CD22,CD34,CD44
3010	Ribosome	Genetic Information Processing	Translation	47	134	0.350746	1.30E-07	9.13E-06	RPL36A-HNRNPH2,RPS10-NUDT3,RPL35,RPL10L,RPL22L1,RPL36,RPS27L,RPL3L,RPL9,RPL10,RPL11,RPL12,RPL17,RPL18,RPL23A,RPL24,RPL27,RPL30,RPL27A,RPL28,RPL29,RPL31,RPL32,RPL34,RPL35A,RPL36AL,RPL37,RPL37A,RPL38,RPL39,RPL36A,RPLP2,RPS3A,RPS8,RPS12,RPS13,RPS14,RPS15A,RPS17,RPS1

									8,RPS21,RPS24,RPS25,RPS27,RPS27A,RPS28,RPS29
4979	Cholesterol metabolism	Organismal Systems	Digestive system	24	50	0.48	2.33E-07	1.36E-05	CETP,CYP27A1,ABCA1,PCSK9,ANGPTL3,MYLIP,APOA1,APOA2,APOA4,APOB,APOC1,APOC2,APOC3,APOE,APOH,LCAT,LIPC,ABCG5,ABCG8,STAR,TSPO,SOAT2,LIPG,SCARB1
4974	Protein digestion and absorption	Organismal Systems	Digestive system	38	103	0.368932	4.82E-07	2.41E-05	KCNE3,COL1A1,COL1A2,COL3A1,COL4A1,COL4A2,COL5A1,COL5A2,COL6A1,COL6A2,COL6A3,COL8A2,COL9A3,COL11A2,COL13A1,COL15A1,COL16A1,COL19A1,CPA1,CPB2,DPP4,SLC6A19,KCNQ1,MEP1A,MME,ATP1A2,ATP1B2,PRSS3,COL20A1,SLC1A5,SLC3A1,SLC15A1,COL14A1,COL18A1,COL21A1,KCNK5,SLC7A7,COL23A1
5144	Malaria	Human Diseases	Infectious disease: parasitic	22	50	0.44	4.60E-06	2.02E-04	COMP,CR1L,GYPA,GYPB,GYPC,HBA1,HBA2,HGF,ICAM1,IL18,ITGAL,ITGB2,MYD88,PECAM1,TLR9,CCL2,SELP,TGFB1,TGFB2,TLR2,TLR4,CD40
4151	PI3K-Akt signaling pathway	Environmental Information Processing	Signal transduction	91	354	0.257062	5.50E-06	2.14E-04	C8orf44-SGK3,SGK2,LPAR6,CDKN1A,LAMC3,CHAD,CHRM2,COL1A1,COL1A2,COL4A1,COL4A2,COL6A1,COL6A2,COL6A3,COL9A3,COMP,CSF1,CSF1R,PIK3R6,CREB3L4,EFNA1,EIF4EBP1,EPOR,ERBB3,FGF4,FG

									FR3,FGFR4,VEGFD,ITGA11,FLT1,FLT3LG,FLT4,FN1,PIK3R5,LPAR3,GHR,GNB3,GNG11,ANGPT2,HGF,TNC,IGF1,IGF2,IL2RB,IL3RA,IL4R,IL6R,ITGA1,ITGA2B,ITGA5,ITGA9,ITGB3,JAK3,KDR,LAMA2,LAMA5,LAMB1,LAMB3,LAMC1,NOS3,NTF4,NTRK1,OSM,PCK1,PCK2,ANGPT4,PDGFA,PDGFB,PDGFRA,PDGFRB,PGF,DDIT4,PPP2R5A,RELN,GNB4,CREB3L2,TEK,TLR2,TLR4,TNXB,VEGFA,VEGFC,VTN,VWF,FGF23,CREB3L3,ITGA10,ITGA8,CCND2,CREB3L1,FGF19
5323	Rheumatoid arthritis	Human Diseases	Immune disease	33	93	0.354839	7.20E-06	2.53E-04	TCIRG1,TNFSF13B,CSF1,CTSK,FLT1,FOS,ATP6V0D2,HLA-DMA,HLA-DMB,HLA-DOA,HLA-DOB,HLA-DPA1,HLA-DPB1,HLA-DQB1,HLA-DRB1,HLA-DRB3,ICAM1,IL11,IL18,ITGAL,ITGB2,MMP1,ACP5,CCL2,CXCL12,TEK,TGFB1,TGFB2,TLR2,TLR4,VEGFA,TNFSF13,CD80
4514	Cell adhesion molecules	Environmental Information Processing	Signaling molecules and interaction	46	149	0.308725	9.94E-06	3.17E-04	CDH3,CDH5,CDH15,CLDN23,VCAN,CLDN19,HLA-DMA,HLA-DMB,HLA-DOA,HLA-DOB,HLA-DPA1,HLA-DPB1,HLA-DQB1,HLA-DRB1,HLA-DRB3,HLA-E,ICAM1,ICAM2,ICAM3,ITGA9,ITGAL,ITGAM,ITGB2,CD99,MPZ,CLDN11,F11R,PE

									CAM1,PVR,SDC4,SELP,SELPLG,VSIR,SIGLEC1,SPN,CLDN5,VTCN1,ITGA8,ESAM,CD4,CD6,CD8B,CD22,CD80,CD34,CD40
4020	Calcium signaling pathway	Environmental Information Processing	Signal transduction	66	240	0.275	1.09E-05	3.19E-04	CHRM2,ADCY7,PLCD3,CHRM3,ADORA2A,ADORA2B,ADRB3,EDNRA,ADCY4,ERBB3,FGF4,FGFR3,FGFR4,VEGFD,FLT1,FLT4,GDNF,GRIN2A,GRIN2D,GRPR,HGF,HRC,HRH2,HTR6,HTR7,ITPR1,ITPR2,ITPR3,KDR,MST1,MST1R,NOS3,ATP2A1,ATP2A3,NTRK1,ATP2B3,P2RX1,PLCE1,PDGFA,PDGFB,PDGFRA,PDGFRB,PLCB2,PLCD1,TPCN1,MCOLN3,CYSLTR2,BDKRB1,BDKRB2,TACR1,TBXA2R,TNNC2,TNNC1,VEGFA,VEGFC,CACNA1F,FGF23,CAMK2A,CASQ1,PLCD4,ORAI1,CCKBR,P2RX6,MYLK3,GNA14,FGF19
4080	Neuroactive ligand-receptor interaction	Environmental Information Processing	Signaling molecules and interaction	89	353	0.252125	1.59E-05	4.30E-04	APELA,LPAR6,CALCRL,NMUR1,CGA,GPR83,UTS2,CHRM2,CHRM3,CHRNA3,CHRND,CHRNE,CHRNG,GRIN3B,LTB4R,LYPD6B,ADORA2A,ADORA2B,ADRA2B,ADRA2C,ADRB3,GPR156,AGT,AGTR2,APLNR,S1PR3,EDN3,EDNRA,F2,F2RL1,F2RL2,LPAR3,FPR2,FSHR,GABRA5,GABRE,GABRR2,GCGR,GHR,GIPR,NPW,MCHR1,GRIN2A,GRIN2D,NR3C1,GRM4,GRPR,HRH2,HTR1D,HTR6,HTR7,IAPP,INSL3,KISS1,K

									NG1,LEPR,LHB,MC1R,NTS,OPRD1,OXT,P2RX1,P2RY1,P2RY6,GAL,PLG,S1PR5,POMC,PRSS3,PYY,CYSLTR2,PTGDR,PTGER2,PTGER4,PTGIR,PTH1R,RLN2,BDKRB1,BDKRB2,SST,TAC3,TACR1,TBXA2R,TSPO,C3,C5AR1,ADM2,CCKBR,P2RX6
5150	Staphylococcus aureus infection	Human Diseases	Infectious disease: bacterial	32	95	0.336842	3.31E-05	8.31E-04	DEFB1,KRT24,FCGR2A,FGG,FPR2,HLA-DMA,HLA-DMB,HLA-DOA,HLA-DOB,HLA-DPA1,HLA-DPB1,HLA-DQB1,HLA-DRB1,HLA-DRB3,ICAM1,CFI,ITGAL,ITGAM,ITGB2,KRT14,KRT16,KRT19,PLG,CFB,SELP,SELPLG,C1R,C1S,C2,C3,C4B,C5AR1
4060	Cytokine-cytokine receptor interaction	Environmental Information Processing	Signaling molecules and interaction	75	295	0.254237	5.51E-05	0.001289	TNFSF13B,EDAR,CCR1,IL17RE,CSF1,CSF1R,CSF2RA,CSF2RB,CX3CR1,EDA,EPOR,CLCF1,AMHR2,GHR,IL17B,BMP10,CCR10,IL1R1,IL1RAP,IL2RB,IL3RA,IL4R,IL6R,IL6ST,IL9R,IL10RA,IL11,IL11RA,IL16,IL18,GDF6,LEPR,LIF,LIFR,LTBR,MPL,NODAL,OSM,PF4,IL20RA,PPBP,IL17RB,IL36G,IL22RA1,EDA2R,CCL2,CXCL12,BMP4,BMP5,TGFB1,TGFB2,THPO,TNFRSF1B,TNFSF4,INHBE,TNFSF13,TNFSF12,TNFSF10,TNFSF9,TNFRSF10D,TNFRSF10C,TNFRSF10B,TNFRSF10A,IL18R1,IL33,CD4,IL

									32,ACVRL1,TNFRSF8,IL27RA,GDF15,GDF3,CD40,CD70,TNFSF15
4510	Focal adhesion	Cellular Processes	Cellular community - eukaryotes	55	201	0.273632	6.77E-05	0.001398	LAMC3,MYL9,BUB1B-PAK6,CHAD,COL1A1,COL1A2,COL4A1,COL4A2,COL6A1,COL6A2,COL6A3,COL9A3,COMP,VEGFD,ITGA11,FLNC,FLT1,FLT4,FN1,PARVB,HGF,BIRC3,TNC,IGF1,ITGA1,ITGA2B,ITGA5,ITGA9,ITGB3,KDR,LAMA2,LAMA5,LAMB1,LAMB3,LAMC1,PDGFA,PDGFB,PDGFRA,PDGFRB,PGF,RELN,PAK6,MYL7,RAC2,SHC1,TNXB,VAV1,VEGFA,VEGFC,VTN,VWF,ITGA10,ITGA8,CCND2,MYLK3
4611	Platelet activation	Organismal Systems	Immune system	38	124	0.306452	6.90E-05	0.001398	RASGRP2,ADCY7,COL1A1,COL1A2,COL3A1,PIK3R6,ADCY4,F2,FCER1G,FCGR2A,FGA,FGB,FGG,PIK3R5,GP1BA,GP9,GUCY1A1,ITGA2B,ITGB3,ITPR1,ITPR2,ITPR3,LCP2,LYN,NOS3,P2RX1,P2RY1,PLA2G4A,PLCB2,APBB1IP,PTGIR,TBXA2R,TBXAS1,BTK,VWF,FERMT3,ORAI1,MYLK3
5410	Hypertrophic cardiomyopathy	Human Diseases	Cardiovascular disease	30	90	0.333333	7.22E-05	0.001398	DES,AGT,ITGA11,IGF1,ITGA1,ITGA2B,ITGA5,ITGA9,ITGB3,LAMA2,MYH6,MYL3,ATP2A1,ATP2A3,PRKAB2,SGCA,SGCG,ACTC1,TGFB1,TGFB2,TNNC1,TNNI3,TNNT2,TPM1,TPM2,CACNA1F,CACNB2,ITGA10,ITGA8,CACNA2D4

4975	Fat digestion and absorption	Organismal Systems	Digestive system	18	43	0.418605	7.57E-05	0.001398	AGPAT2,ABCA1,FABP1,NPC1L1,APOA1,APOA4,APOB,MOGAT3,MTTP,PLA2G3,PLA2G2A,PNLIPRP2,ABCG5,ABCG8,PLA2G12B,DGAT2,PLPP2,SCARB1
5414	Dilated cardiomyopathy	Human Diseases	Cardiovascular disease	31	96	0.322917	1.08E-04	0.001899	ADCY7,DES,AGT,ADCY4,ITGA11,IGF1,ITGA1,ITGA2B,ITGA5,ITGA9,ITGB3,LAMA2,MYH6,MYL3,ATP2A1,ATP2A3,SGCA,SGCG,ACTC1,TGFB1,TGFB2,TNNC1,TNNI3,TNNT2,TPM1,TPM2,CACNA1F,CACNB2,ITGA10,ITGA8,CACNA2D4
